# Entrepreneurial Psychological Quality and Quality Cultivation of College Students in the Higher Education and Moral Education Perspectives

**DOI:** 10.3389/fpsyg.2021.700334

**Published:** 2021-09-01

**Authors:** Qinghua Cao

**Affiliations:** Center for Ideological and Political Education, Northeast Normal University, Changchun, China

**Keywords:** entrepreneurial psychological quality of college students, entrepreneurship education for college students, entrepreneurial traits, entrepreneurial self-efficacy, entrepreneurial alertness, entrepreneurial attitude

## Abstract

The present work aims to explore the psychological quality and quality of college students' entrepreneurship under higher education and moral education. First, relationships among entrepreneurial traits, entrepreneurial self-efficacy, entrepreneurial alertness, and entrepreneurial attitude are analyzed through questionnaire surveys and statistics. Second, the role of college students' entrepreneurial attitudes in improving entrepreneurial self-efficacy and entrepreneurial alertness is discussed. Eventually, the relationship mechanism of entrepreneurial self-efficacy and entrepreneurial alertness in entrepreneurial traits and entrepreneurial attitude is explored. Results demonstrate: (1) the mediating effect through the entrepreneurial self-efficacy reaches 36.91%; (2) the mediating effect through entrepreneurial alertness accounts for 38.72%; (3) the mediating effect through entrepreneurial self-efficacy and entrepreneurial alertness reaches 9.15%. Therefore, the entrepreneurial traits of college students affect their entrepreneurial attitude through two intermediary paths: entrepreneurial self-efficacy and entrepreneurial alertness. Data comparison suggests that the entrepreneurial traits of college students are significantly positively correlated to entrepreneurial attitudes; the entrepreneurial self-efficacy of college students is significantly positively correlated to entrepreneurial attitude; the entrepreneurial alertness of college students is significantly positively correlated to entrepreneurial attitudes. College students' entrepreneurial self-efficacy plays a mediating role in the relationship between entrepreneurial traits and entrepreneurial attitude, and college students' entrepreneurial alertness plays a mediating role in the relationship between entrepreneurial traits and entrepreneurial attitude.

## Introduction

Under the accelerated development of the market economy and knowledge economy, China encourages and promotes college graduates to start their businesses and has introduced a series of preferential policies for this purpose (Yan et al., 2018). Various colleges and universities have also launched entrepreneurship competitions. Many parents also encourage their children to start businesses. All the above phenomena have inspired college student entrepreneurs (Yin and Wang, 2017; Isabelle, 2020). However, in general, most students dare not or are not good at starting a business due to not only the psychological quality of themselves but also the family and society (Pandit et al., 2018; Jena, 2020). Psychological quality refers to the comparatively stable characteristics of people in terms of psychology. It consists of several interrelated elements, such as cognition, will, emotion, and personality. The psychological quality of entrepreneurs refers to the psychological conditions of entrepreneurs, including personality, feelings, self-awareness, temperament, and other psychological components (Sang and Lin, 2019; Wu et al., 2019).

Wu et al. (2019) evaluated the experience of students using mobile-based CRS technology in entrepreneurship courses. They found that mobile-based CRS technology could promote the interaction between learners and content and improve students' participation in entrepreneurial knowledge acquisition, which served as an effective tool to improve the psychological motivation of students' entrepreneurial ability. In particular, regardless of time and location, students could experience innovative, active, and in-depth learning in mobile-based and CRS-supported classrooms (Wu et al., 2019). Wu and Song (2019) explored the application and satisfaction of social media in entrepreneurship courses from the perspective of learners. They found that online entrepreneurship groups had four satisfaction factors, namely trust, profit, learning, and social interaction. The two factors of trust and profit could be regarded as the specific satisfaction of the network entrepreneurial group, especially the trust factor, deserving attention in the further research of the social media network entrepreneurship course (Wu and Song, 2019). Obschonka et al. (2018) found that entrepreneurial thinking and entrepreneurial behavior played increasingly important roles in the success of organizations. Entrepreneurial passion was an emerging key structure in organizational behavior research. Theoretical models found that a person's basic entrepreneurial personality characteristics could contribute to the formation of entrepreneurial enthusiasm, associated with actual entrepreneurial activities (Obschonka et al., 2018).

Current works on student entrepreneurship focus on cultivating entrepreneurial psychology and enthusiasm. In the present work, the relationship between entrepreneurial traits and attitude that college students need to possess is analyzed, and the mediating relationship between self-efficacy and alertness is discussed. Hypotheses based on related theories are verified through questionnaire surveys, providing effective support for college students' entrepreneurial education and the cultivation of entrepreneurial psychological quality. The point of innovation is to propose a chain mediating mechanism between entrepreneurs' self-efficacy and entrepreneurial alertness, which can effectively enhance college students' opportunistic entrepreneurial intentions. Also, the social cognition theory and cognitive psychology theory are combined to explain the research mechanism that enhances opportunistic entrepreneurial intentions more reasonably (Liu and Chen, 2021; Liu et al., 2021).

## Theoretical Model Establishment and Research Scheme Design

### Cultivation of Students' Entrepreneurial Psychological Quality Under Higher Education

Schools shoulder the burden of cultivating talents for society, and China's great cause of revitalization requires a large number of talents who can start businesses, have lofty ideals, and possess excellent will and quality. Therefore, college education has become the core of entrepreneurship education for college students to successfully prepare for their businesses, and the cultivation of entrepreneurial psychology has been strengthened. On the one hand, the difficulty of entrepreneurship determines that entrepreneurial psychology is the key to success (Ghafar, 2020). At the beginning of any business, it is impossible to avoid all kinds of difficulties and obstacles, especially to study areas that no one has ever been involved in. Hence, a firm will and sufficient confidence are required to stick to the business and ultimately succeed. If colleges and universities want their students to achieve their careers in life, they must cultivate the psychological quality necessary for their graduates to start their businesses. On the other hand, college students are immature, making colleges and universities the key to cultivating their entrepreneurial psychological quality.

College students are a special group full of vitality and vigor in society; they are the hope of the country and the nation and shoulder the mission and responsibilities entrusted by the new era. Their mind is more open, their vision is broader, and their willingness to challenge themselves is stronger. In general, college students would like to try new things and are more adventurous (Suacamram, 2019). China advocates investment in innovation and entrepreneurship activities of the whole people. Under such a background, college students participate in and become a vital link of the entrepreneurship activities. The definition of entrepreneurship by college students refers to the process by which those who are good at capturing opportunities and daring to try can identify the development opportunities, integrate and utilize surrounding resources, and transform their entrepreneurial ideas into practical actions under the careful consideration of their abilities and interests. The purpose of entrepreneurship is to ensure employment while achieving individual value (You et al., 2017). The social cognition theory was originally based on the social learning theory with observational learning as the central point of view. The social learning theory forms the overall framework of the social cognition theory together with the ternary interaction and self-efficacy theories, laying a significant theoretical foundation for entrepreneurial research.

The relationship between the entrepreneurial traits possessed by college students and their entrepreneurial attitude is explored from the perspective of higher education and moral education. The entrepreneurial self-efficacy and entrepreneurial alertness are taken as mediating variables to investigate the relationship model and determine the research hypotheses. The relationships among key variables are tested using the questionnaire survey and statistical analysis methods, and the mechanism of cultivating college students' entrepreneurial psychological quality is discussed. Influences of college students' key entrepreneurial cognitive elements, that is, entrepreneurial self-efficacy and entrepreneurial alertness, on the relational mechanism of entrepreneurial traits and entrepreneurial attitude are researched based on the social cognition theory. The results can provide practical references and data support for college students' entrepreneurship education and entrepreneurial psychological quality cultivation.

### Establishment of Ternary Interaction Model

**Figure 1:**
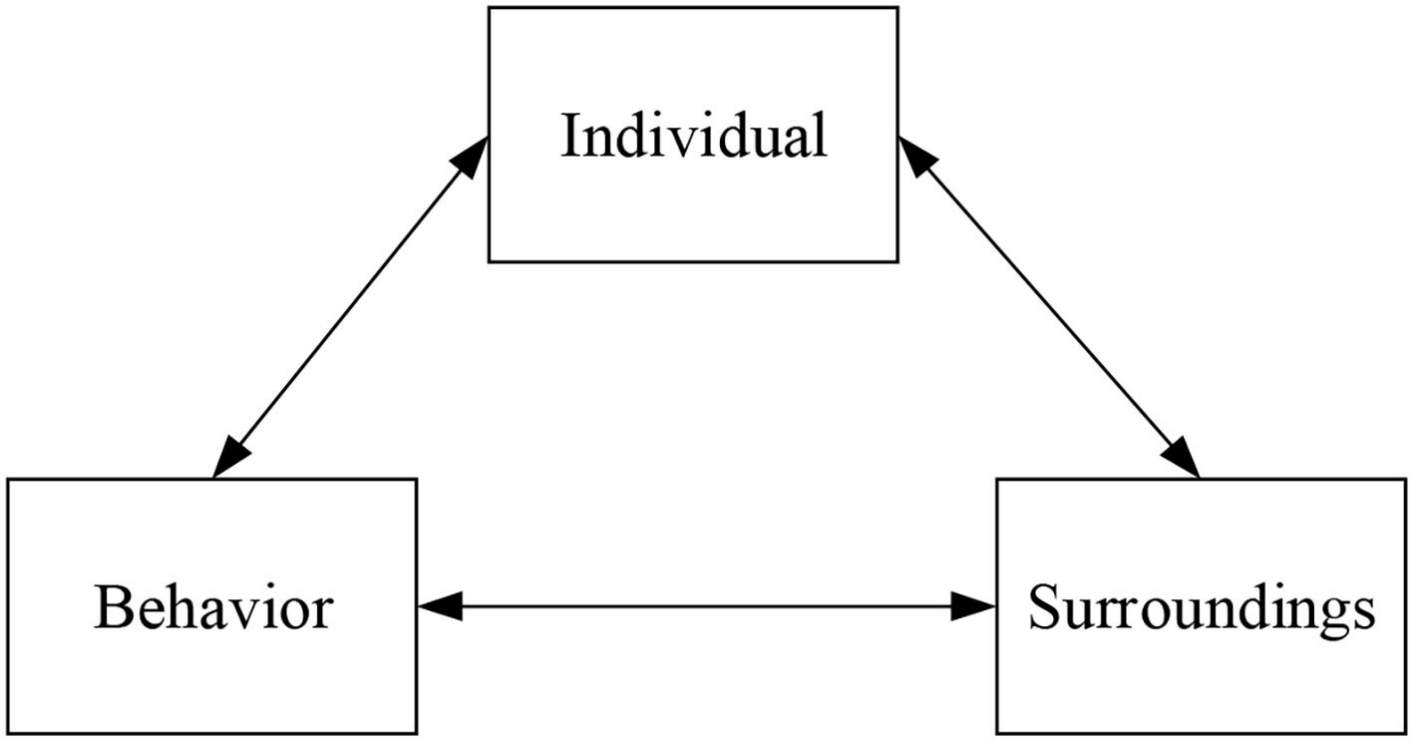
Ternary interaction model.

Observational learning theory believes that an individual who learns a behavior does not necessarily have to obtain direct practical experience through practical operations. Instead, the self-recognition of a behavior can be achieved by observing social models or typical practices around this individual, thereby learning the experience of this behavior and forming the correct way of thinking or behavior. The self-efficacy theory refers to the self-estimation and judgment of individuals on whether they can conduct specific behaviors, reflecting the self-confidence of individuals in their abilities. Self-efficacy is individuals' subjective estimation of whether they can complete a task or activity rather than possessing this ability. When individuals complete or perform different tasks, their subjective evaluations of their abilities are different. Self-efficacy is a kind of strong personal subjective belief based on the premise that individuals have a comprehensive perception and understanding of all aspects of their abilities (Wang et al., 2019). In the ternary interaction theory, individual factors refer to individuals' perception of their characteristics and self-cognition of the occurrence of actions. Behavioral factors refer to the sum of observable action elements such as the individual's social actions, responses to the outside world, and willingness and motivation. These factors are affected by the external environment and are associated with individuals' perceptions of their knowledge, experience, and behavior (Liguori et al., 2018). The interaction between these three elements becomes the core component of the ternary interaction model, as shown in Figure 1 below.

The social cognition theory provides a dynamic and comprehensive perspective for studying the generation and development of individual behavior. In general, individual behavior is not only a concrete manifestation of personal characteristics but also the result of external environmental influences (Li and Wu, 2019). Observational learning emphasizes the demonstrative role of external role models around the individuals and the important role of the environment. Self-efficacy emphasizes the individual's internal behavioral beliefs and focuses on the influence of individual characteristics (Garcia et al., 2019). The ternary interaction theory incorporates the individual's behavior, the individual itself, and the external environment into a dynamic influence model, focusing on the combined effect of the three elements. The social cognitive theory comprehensively analyzes the mechanism of the generation and development of behavior, laying a solid theoretical foundation for the research on college students' entrepreneurial psychological quality and quality cultivation (Darmanto and Yuliari, 2018).

### Analysis of College Students' Entrepreneurial Behavior Intention Model

Entrepreneurship, an activity with multiple attractive values, is in line with the professional concepts and value cognition held by college students. They can see the value behind this form of activity, explaining college students choosing entrepreneurship from a personal perspective (Wang et al., 2018). Mair researched the formation process of college students' entrepreneurship and pointed out that the formation of this behavior was a complex process of psychological cognition, subject to the combined effects of individual-associated psychological and behavioral characteristics and environmental factors (Bloemen-Bekx et al., 2019). Therefore, the social cognitive theory's research on the mechanism of behavior generation provides a comprehensive perspective for further exploring the formation process of college students' entrepreneurial psychological quality (Chen, 2018, 2019).

**Figure 2:**
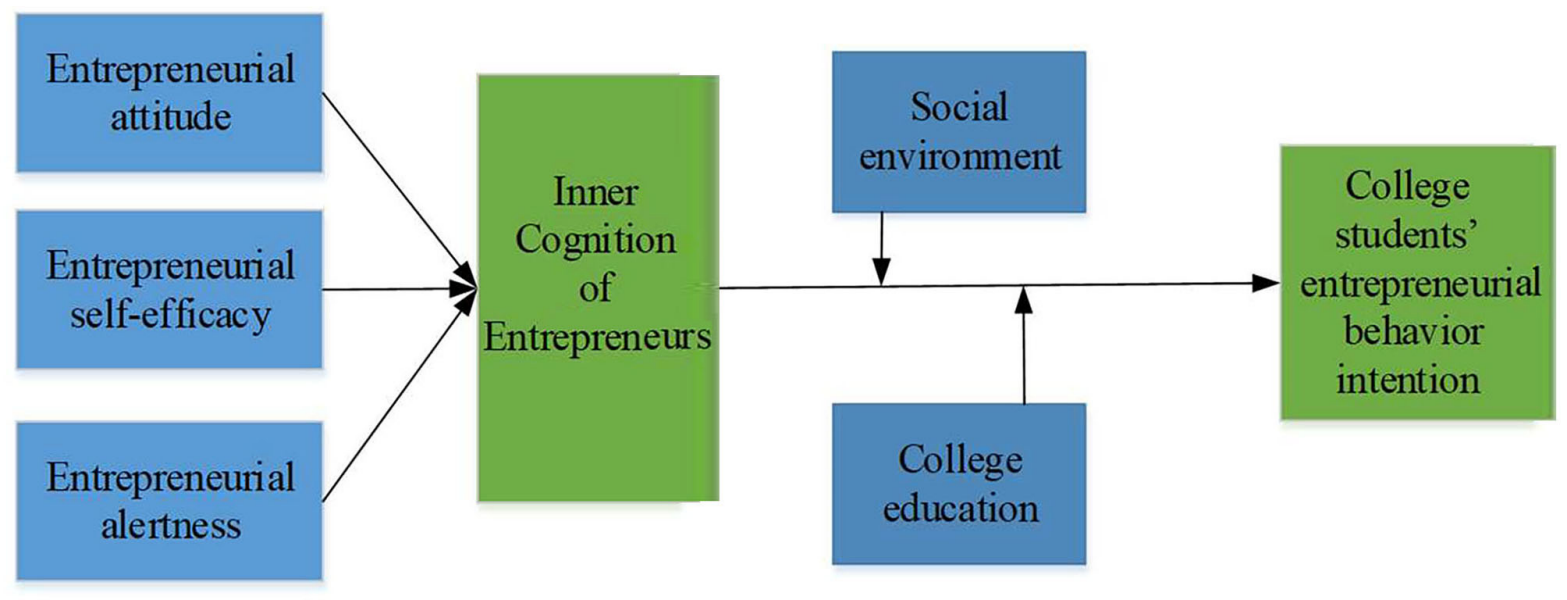
College students' entrepreneurial behavior intention model.

College students' psychological perception of entrepreneurship directly determines whether their entrepreneurial willingness is generated or not. On the one hand, students' emotions, attitudes, and other perceptual factors affect their perception of entrepreneurial value; that is, whether it is necessary to do such kind of entrepreneurial activity (Meoli et al., 2020). Students' quality and sense of efficacy can affect their perception of their abilities to participate in entrepreneurial activities; that is, whether they are capable of starting a business. Moreover, while the force from the external environment provides students with entrepreneurial support, it will also affect their views on entrepreneurship, which, in turn, affects their entrepreneurial willingness. For college students, the more they believe in the value of entrepreneurial behavior of entrepreneurship, the more confident they are in their abilities and qualities, the more complete the entrepreneurial support from the surrounding environment, the stronger their willingness to start a business, and the more likely they are to perform specific entrepreneurial behaviors (Mair and Marti, 2006). Based on the above analyses, a framework of the formation process of college students' entrepreneurial psychological quality is constructed, as shown in Figure 2 below.

### Influencing Factor Model of College Students' Entrepreneurial Psychological Quality

According to the social cognition theory, as the initiator and promoter of specific behavior, many factors of the individuals have a direct impact on their behaviors. Regarding the specific entrepreneurial behaviors, scholars have also identified the impact of entrepreneurship. Darmanto's quantitative research on entrepreneurship concluded that the background characteristics and personality characteristics of individuals, such as gender, age, and educational background, were important factors for predicting entrepreneurial willingness (Kucher et al., 2019; Liu et al., 2019).

Therefore, the individual is one of the key variables that determine the generation and development direction of entrepreneurial activities. As an emerging force in entrepreneurship, college students are a special entrepreneurial group that deserves attention, and the study of their characteristics is also very necessary (Hasan et al., 2020). It is believed that college students' psychological and behavioral characteristics and their attitudes and emotions toward entrepreneurship can affect their value judgments on entrepreneurship.

In contrast, their perception of their entrepreneurial abilities and their matching characteristics with entrepreneurship can affect their confidence in entrepreneurship. The above factors ultimately determine whether students have the psychological quality of entrepreneurship. According to the analysis of individual characteristics worldwide, four important variables of entrepreneurial traits, entrepreneurial alertness, entrepreneurial self-efficacy, and entrepreneurial attitude, are selected to measure the characteristics of entrepreneurial psychology and behavior of college students (Feng and Chen, 2020).

A large number of investigations have proved that the entrepreneurial support provided by colleges and universities plays an essential role in promoting students' entrepreneurial willingness. Krueger's research on the entrepreneurial environment actively constructed by universities found that the entrepreneurial support system formed by universities can enhance students' self-awareness of entrepreneurship. The more complete the support system, the more entrepreneurial knowledge students would learn, and the more that students believed they could successfully start a business (Apriana et al., 2019). While college students transform their identity from students to entrepreneurs, the entrepreneurial support provided by colleges and universities meets their vision of entrepreneurship, cultivates their entrepreneurial psychological quality, and stimulates their entrepreneurial willingness.

The entrepreneurial support system of colleges and universities incorporates the concepts of ethics, responsibility, and sustainable development into the teaching content, forming a diversified cooperation system with public departments and social enterprises, which can help students form a correct understanding of entrepreneurship and realize the value and feasibility of entrepreneurship. Ultimately, the identity of entrepreneurs can be strengthened, thereby promoting the cultivation of college students' entrepreneurial psychological quality.

### Analysis of Students' Entrepreneurship Ability Based on Entrepreneurship

College students who devote themselves to entrepreneurial activities are essentially entrepreneurs. Research on entrepreneurship has shown that successful entrepreneurs have similar personal characteristics to traditional entrepreneurs, such as the need for achievement, the spirit of innovation, and the motivation to solve social conflicts. However, these characteristics are only caused by the type of entrepreneurial activity and the pursuit of entrepreneurship. The ultimate goal of different entrepreneurs is different, and there are also differences in the personal characteristics shown by different entrepreneurs.

Under the current severe employment situations, the reason that entrepreneurs among college students choose to start a business is that they are more daring to take risks, have a stronger need for achievement, and have a spirit of innovation. These characteristics also drive them to produce the idea of starting a business. Academia generally believes that when the characteristics of an individual match the qualities required for entrepreneurship, the greater the possibility of generating entrepreneurial ideas, the greater the probability of engaging in entrepreneurial activities.

Entrepreneurial alertness is an ability. Kirzner defined entrepreneurial alertness as an individual's ability to have a keen sense of potential information. Entrepreneurial alertness is a special perception of entrepreneurial information. Such information includes market information, entrepreneurial resource information, and industry development trends (Jemal, 2017). In addition, entrepreneurial alertness also refers to the ability to further explore entrepreneurial opportunities that others have discovered. Individuals with entrepreneurial alertness can easily identify potential opportunities and do not need to search for opportunities deliberately; thus, they can analyze the feasibility of business opportunities. Second, entrepreneurial alertness is a mental model.

In this model, individuals are keenly aware of entrepreneurial information and can judge entrepreneurial opportunities. Entrepreneurial alertness is divided into three types of alertness based on individual mental models. Perceived alertness emphasizes the alertness of individuals to obtain various types of entrepreneurial information. Thinking alertness refers to the alertness of individuals to process, reorganize, and integrate various types of entrepreneurial information. Response alertness refers to the attitude of an individual after reorganizing entrepreneurial information (Krueger et al., 2000). Here, entrepreneurial alertness is defined as the cognitive ability of college students to predict the market based on the collected entrepreneurial information. Entrepreneurial alertness is not inborn but is formed based on the knowledge and beliefs possessed by the individual. Its essence is a complicated cognitive framework that is alert to the social environment (Deng et al., 2021).

Entrepreneurial self-efficacy, one of the important concepts in the field of entrepreneurial research, represents individuals' self-perception of their abilities to match specific entrepreneurial tasks and reflects the entrepreneurs' self-confidence degree. Individuals with a strong sense of entrepreneurial self-efficacy are better at grasping the hidden opportunities in the environment, are more optimistic in the face of difficulties, have a better ability to deal with risks, and are more likely to achieve better entrepreneurial results (Wilson et al., 2007).

At the current time when social contradictions are constantly emerging, and employment problems are increasingly prominent, entrepreneurship has great uncertainty. Only if students have enough confidence in their abilities and firmly believe that they can overcome predicted risks and difficulties, that is, students with a higher level of self-efficacy, will they have the idea of starting a business.

### Research Hypotheses

Entrepreneurial attitude reflects individuals' cognition and views on entrepreneurship and the value judgments made by individuals about entrepreneurship. The greater the value of entrepreneurial behavior to individuals, the more likely they will make specific entrepreneurial behaviors. Utami proved that whether the attitude was positive or not directly affected the individual's perception of the entrepreneur's role. When individuals' entrepreneurial attitude was positive, the more they were willing to demand themselves as entrepreneurs, the stronger their willingness to start a business (Kirzner, 2009). Regarding the entrepreneurial practice results in China, choosing to start a business means to assume the dual identities of entrepreneur and social repairperson, which is also a huge challenge for college students who have just entered society. Table 1 below summarizes the five research hypotheses.

**Table 1:** Research hypotheses.

**Hypotheses**	**Contents**
H1:	College students' entrepreneurial traits and entrepreneurial attitude share a significantly positive correlation.
H2:	College students' entrepreneurial self-efficacy and entrepreneurial attitude share a significantly positive correlation.
H3:	College students' entrepreneurial alertness and entrepreneurial attitude share a significantly positive correlation.
H4:	College students' entrepreneurial self-efficacy plays a mediating role in the relationship between entrepreneurial traits and entrepreneurial attitude.
H5:	College students' entrepreneurial alertness plays a mediating role in the relationship between entrepreneurial traits and entrepreneurial attitude.

### Research Model and Survey Analysis Method

All data come from the survey of *College Students' Entrepreneurial Psychological Quality And Entrepreneurial Attitude Questionnaire*. The selected scales all come from the maturity scales of entrepreneurship research. The questionnaire includes the following five parts: demographic variables, entrepreneurial traits, entrepreneurial self-efficacy, entrepreneurial alertness, and entrepreneurial attitude. A total of 1,000 questionnaires were issued, and 975 were returned. Thirty-two invalid ones were kicked out, leaving 943 valid questionnaires, with a response rate of 94.3%. In particular, paper questionnaires totaled 632 copies (67%), and the electronic questionnaires totaled 311 copies (33%). Regarding the gender of samples whose questionnaires are valid, 367 are boys (39%), and 576 are girls (61%). Regarding the grades of samples, there are 193 first-year students (20.5%), 199 second-year students (21.1%), 323 third-year students (34.3%), 136 fourth-grade students (14.4%), 86 master program students (9.1%), and 6 doctoral program students (0.6%).

Questionnaire surveys are conducted online and offline. And participants are college students aging 19–27 years old from more than 20 colleges and universities. On the one hand, the on-site collection is finished inside the schools; thus, subjects and professions can be chosen to avoid repeated investigations. On the other hand, the online platform for questionnaire collection is the Questionnaire Star. The questionnaires were distributed to more than 20 colleges and universities through the joint efforts of tutors, classmates, and individuals. Before filling out the questionnaires, the participants would be informed of the anonymity of the questionnaires and that the survey was only for academic purposes.

**Figure 3:**
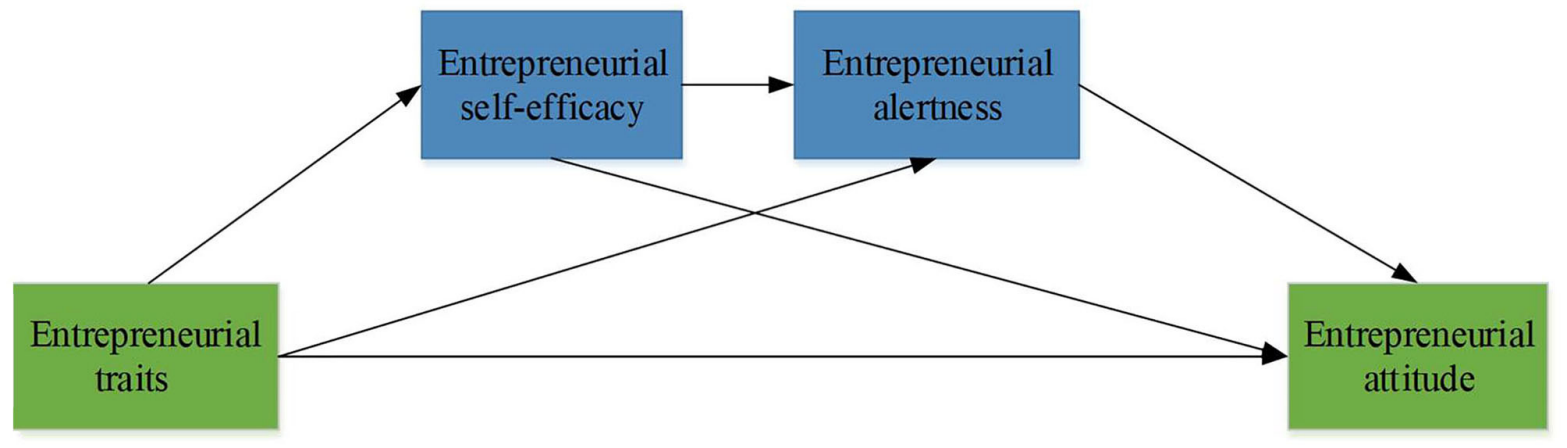
Chained mediation model.

College students' entrepreneurial psychological quality and associated influence mechanisms are researched through questionnaire surveys, and data required for empirical research can be obtained (Newman et al., 2019). In terms of data processing, first, reliability analysis, validity analysis, and correlation analysis are performed on the collected data using SPSS 23.0 software. In addition to the procedural control, the Harman single factor test is also used to exclude the influence of common method deviation. The one-way analysis of variance is performed using SPSS 23.0 to explore the impact of various demographic variables on college students' entrepreneurial psychological quality. Third, a structural equation model is established through AMOS 22.0 to explore the chain effect of entrepreneurial self-efficacy and entrepreneurial alertness between cognitive flexibility and opportunistic entrepreneurial intention, thereby verifying the hypotheses proposed above. Besides, the bias-corrected Bootstrap is performed to test the significance of mediating effect (Soluk et al., 2021). The chained mediating effect of entrepreneurial self-efficacy and entrepreneurial alertness in entrepreneurial traits and entrepreneurial attitude is explored through hypothesis testing and model verification. The chained mediation model is shown in Figure 3.

### The Mediating Effect and Path Analysis of Influencing Factors in College Students' Entrepreneurship

The following econometric models are constructed to test the mediating effect of an individual's entrepreneurial attitude on innovation and entrepreneurship education and college students' entrepreneurial intention.

(1) $$\begin{array}{r} \text{A}I_{i}=\alpha +\beta_{1}AAI_{i}+\beta_{2}B_{i}+\gamma_{i}+\lambda_{i} \end{array}$$

(2) $$\begin{array}{r} MN_{1}=\alpha +\beta_{1}IAA_{i}+\beta_{2}B_{i}+\gamma_{1}+\lambda_{i} \end{array}$$

(3) $$\begin{array}{r} AI_{i}=\alpha +\beta_{1}AAI_{i}+\beta_{2}MN_{i}+\beta_{3}B_{i}+\gamma_{i}+\lambda_{i} \end{array}$$

The model in equation (1) is used to test the direct effect of entrepreneurship education on college students' entrepreneurial intention; The model in equation (2) is used to test the impact of innovation and entrepreneurship education on the entrepreneurial attitude of college students. The model in equation (3) is used to test the mediating effect of influencing factors in college students' entrepreneurship. In the above three equations (1)-(3), A*I*_*i*_ is the entrepreneurial intention, *IAA*_*i*_ is the entrepreneurial level accepted by the sample, *MN*_1_ is the entrepreneurial attitude, and *B*_*i*_ is control variables, including gender, school level, major, grade, entrepreneurial experience, and family self-employment. γ_*i*_ is the influence effect that cannot be observed and λ_*i*_ is the random disturbance term.

**Figure 4:**
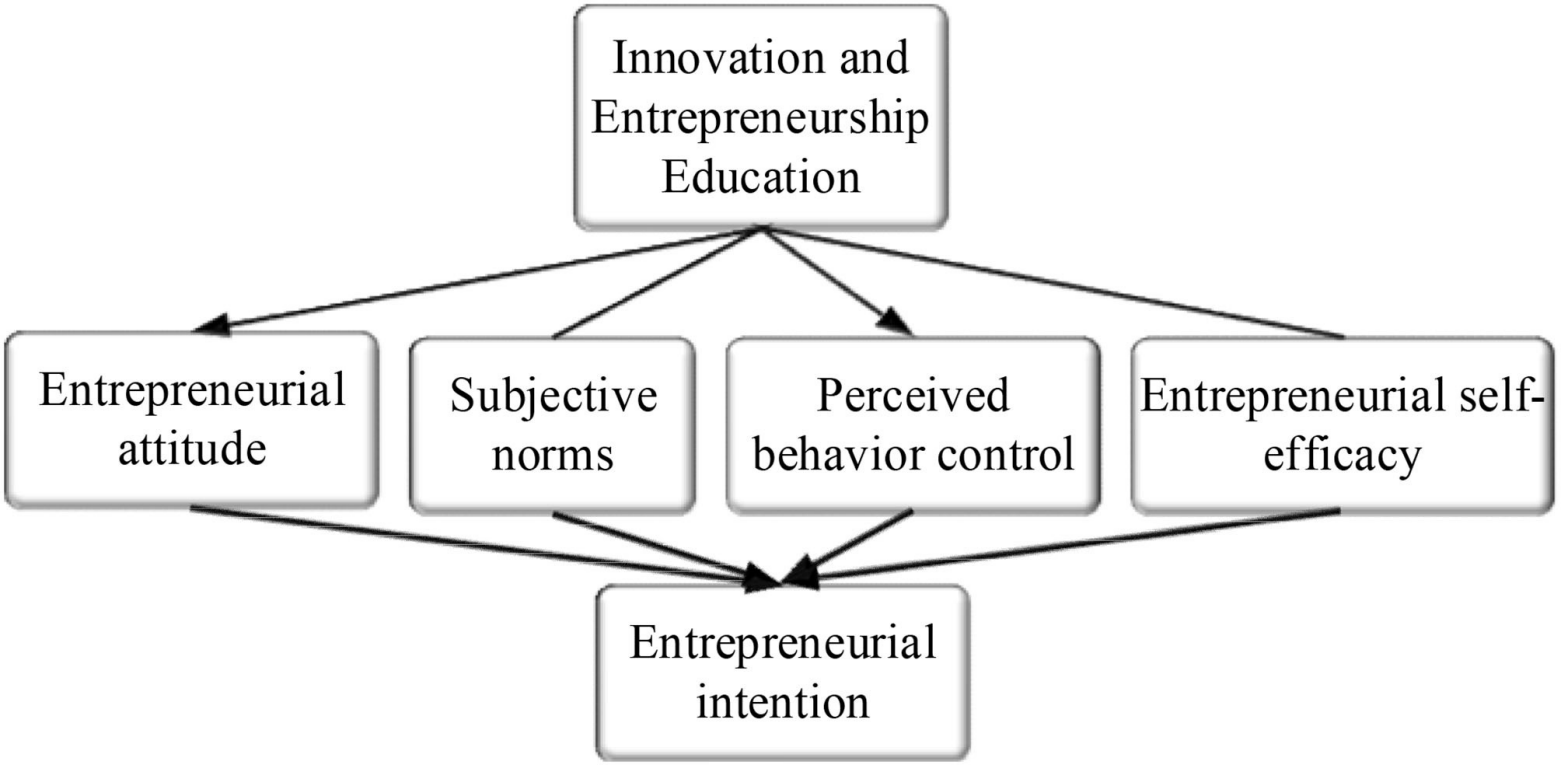
Path map of the mediating effect.

The path map of the mediating effect is shown in Figure 4.

## Results and Discussions

### Analysis of the Questionnaire's Reliability and Validity Results

**Figure 5:**
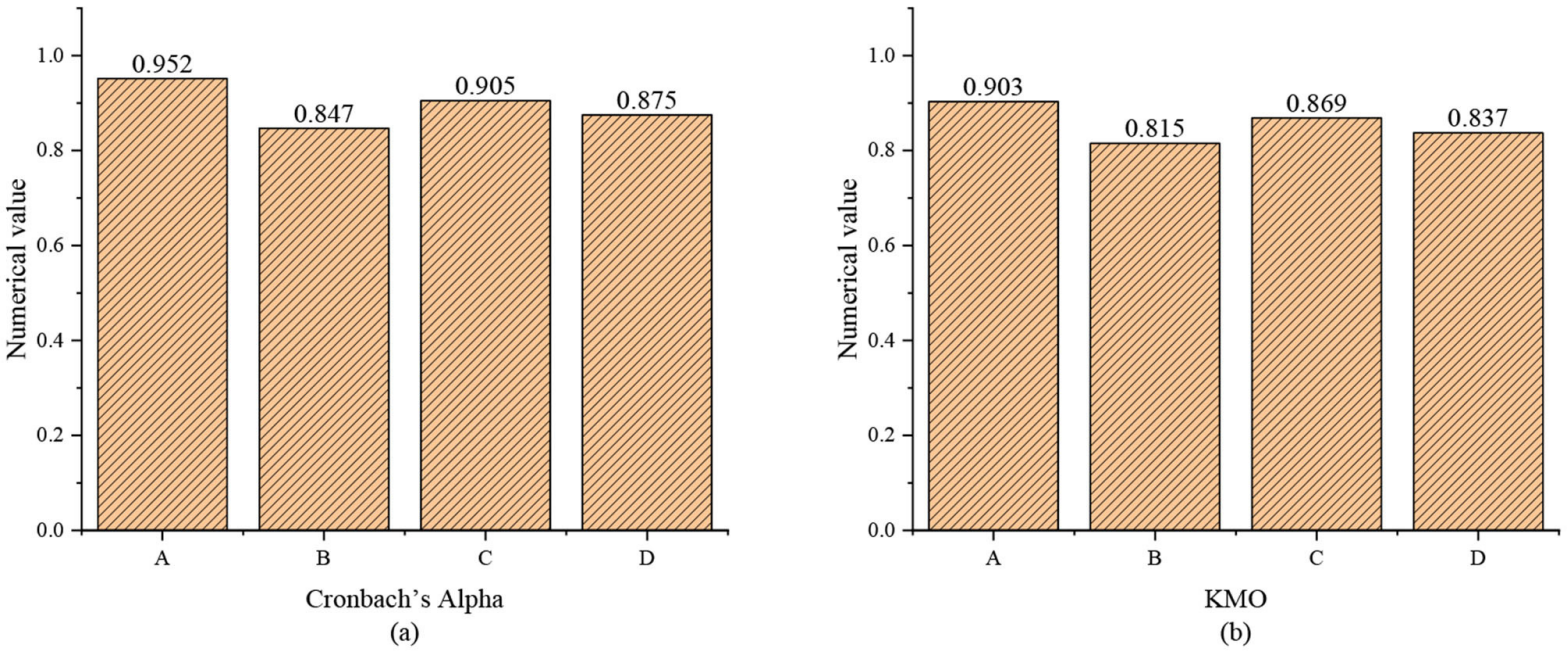
Reliability and validity test of the questionnaire (A: entrepreneurial traits; B: entrepreneurial self-efficacy; C: entrepreneurial alertness; D: entrepreneurial attitude; **(a)** reliability test; **(b)** validity test).

The scales utilized are mature worldwide; hence, they have acceptable validity. The reliability of the questionnaire is tested by the Cronbach's Alpha coefficient method. The larger the coefficient, the higher the reliability of the scale. The validity is tested by the Kaiser-Meyer-Olkin (KMO) measure and Bartlett's test of sphericity. The larger the KMO value, the higher the validity of the scale. The detection and analysis results are summarized in Figure 5.

The SPSS 23.0 software is adopted to analyze the reliability of the sample data. Results show that the Cronbach's Alpha coefficients of the entrepreneurial traits, entrepreneurial self-efficacy, entrepreneurial alertness, and entrepreneurial attitude scales are all >0.8, indicating that the internal consistency of the items on the scales is good. Second, the validity of the sample data is tested to verify the correlation of each item. According to Figure 5, the KMO values of the entrepreneurial traits, entrepreneurial self-efficacy, entrepreneurial alertness, and entrepreneurial attitude scales are all >0.8, indicating that the questionnaire has good validity.

### Descriptive Statistical Analysis of Key Variables

**Figure 6:**
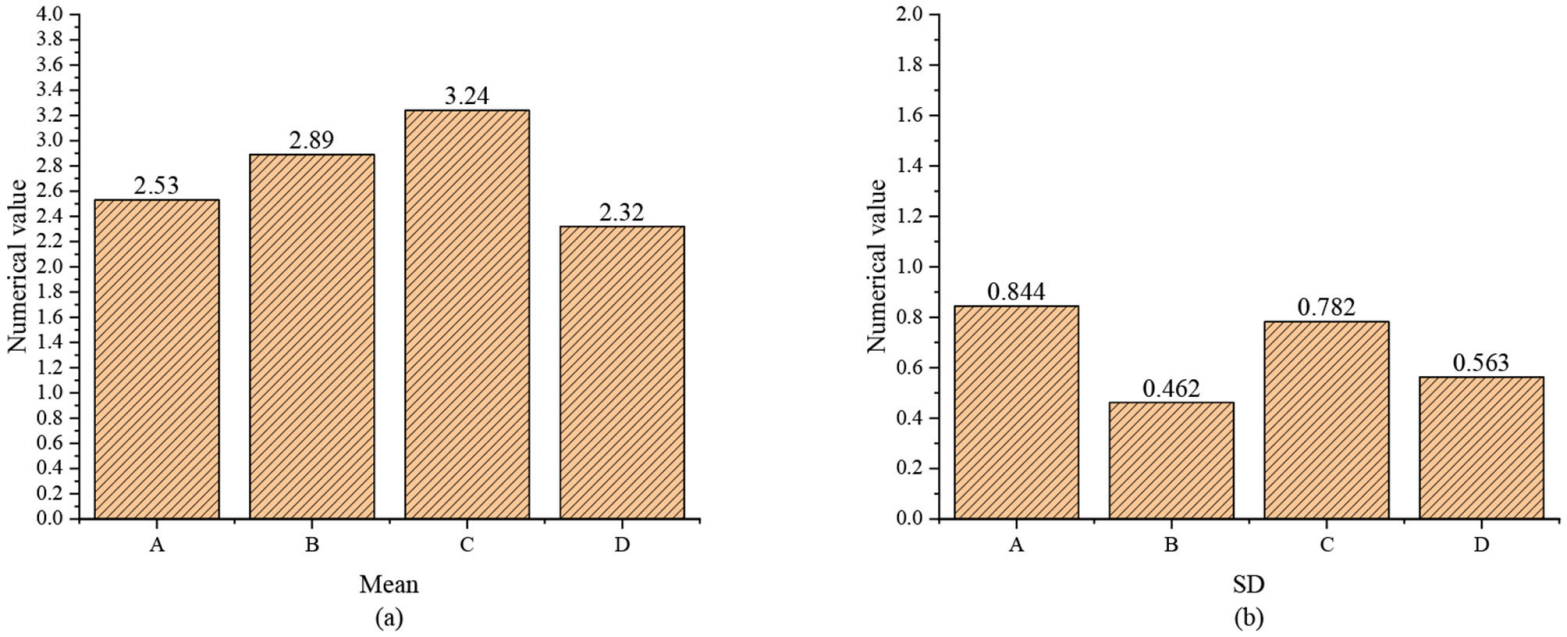
The mean value and standard deviation of key variables (A: entrepreneurial traits; B: entrepreneurial self-efficacy; C: entrepreneurial alertness; D: entrepreneurial attitude; **(a)** mean value; **(b)** standard deviation).

A descriptive statistical analysis of each variable is performed to thoroughly understand the status quo of entrepreneurial traits, entrepreneurial self-efficacy, entrepreneurial alertness, and entrepreneurial attitude of college students. The highest score of the scales is set to 5 points, and the lowest score is set to 1 point. Figure 6 shows the mean value and Standard Deviation (SD) of each variable.

The results suggest that the mean value of college students' entrepreneurial traits is 2.53, <3, indicating that the entrepreneurial traits possessed by college students are not sufficient. The mean value of college students' entrepreneurial alertness is 3.24, indicating that college students have high entrepreneurial alertness. The mean value of college students' entrepreneurial self-efficacy is 2.89, indicating that college students have low confidence in whether they can succeed in difficult and high-risk entrepreneurial activities. The mean value of college students' entrepreneurial attitude is 2.32, indicating that the overall attitude of college students is at a low level.

### Correlation Analysis of Key Variables

The relationships among variables are analyzed through correlation analysis. The results are presented in Table 2.

**Table 2:** Correlation analysis of key variables.

**Dimensions**	**Entrepreneurial traits**	**Entrepreneurial self-efficacy**	**Entrepreneurial alertness**	**Entrepreneurial attitude**
Entrepreneurial traits	1			
Entrepreneurial self-efficacy	0.578^**^	1		
Entrepreneurial alertness	0.485^**^	0.332^**^	1	
Entrepreneurial attitude	0.739^**^	0.647^**^	0.345^**^	1

** *indicates correlation at a significance level of 0.01*.

Table 2 shows the correlation coefficients of entrepreneurial traits, entrepreneurial self-efficacy, entrepreneurial alertness, and entrepreneurial attitude. The above data reveal significant correlations among the four variables. First, the correlation coefficient between entrepreneurial self-efficacy and entrepreneurial traits is 0.578^**^, which passes the significance test, indicating that the higher the entrepreneurial self-efficacy, the stronger the entrepreneurial traits. The correlation coefficient between entrepreneurial alertness and entrepreneurial traits is 0.485^**^, which passes the significance test, indicating that the higher the entrepreneurial alertness, the stronger the entrepreneurial traits. The correlation coefficient between entrepreneurial alertness and entrepreneurial self-efficacy is 0.332^**^, passing the significance test.

Hence, entrepreneurial alertness is positively correlated with entrepreneurial self-efficacy. The higher the entrepreneurial alertness, the higher the level of entrepreneurial self-efficacy. The correlation coefficient between entrepreneurial attitude and entrepreneurial traits is 0.739^**^, passing the significance test. Thus, the stronger the entrepreneurial attitude, the stronger the entrepreneurial traits. The correlation coefficient between entrepreneurial attitude and entrepreneurial self-efficacy is 0.647^**^, which passes the significance test, indicating that the stronger the individual entrepreneurial attitude, the higher the entrepreneurial self-efficacy. Finally, the correlation coefficient between entrepreneurial attitude and entrepreneurial alertness is 0.345^**^, passing the significance test; that is, the stronger the entrepreneurial attitude, the higher the entrepreneurial alertness. The results reveal significant correlations among entrepreneurial traits, entrepreneurial self-efficacy, entrepreneurial alertness, and entrepreneurial attitude.

### Mediating Effect and Path Analysis

**Figure 7:**
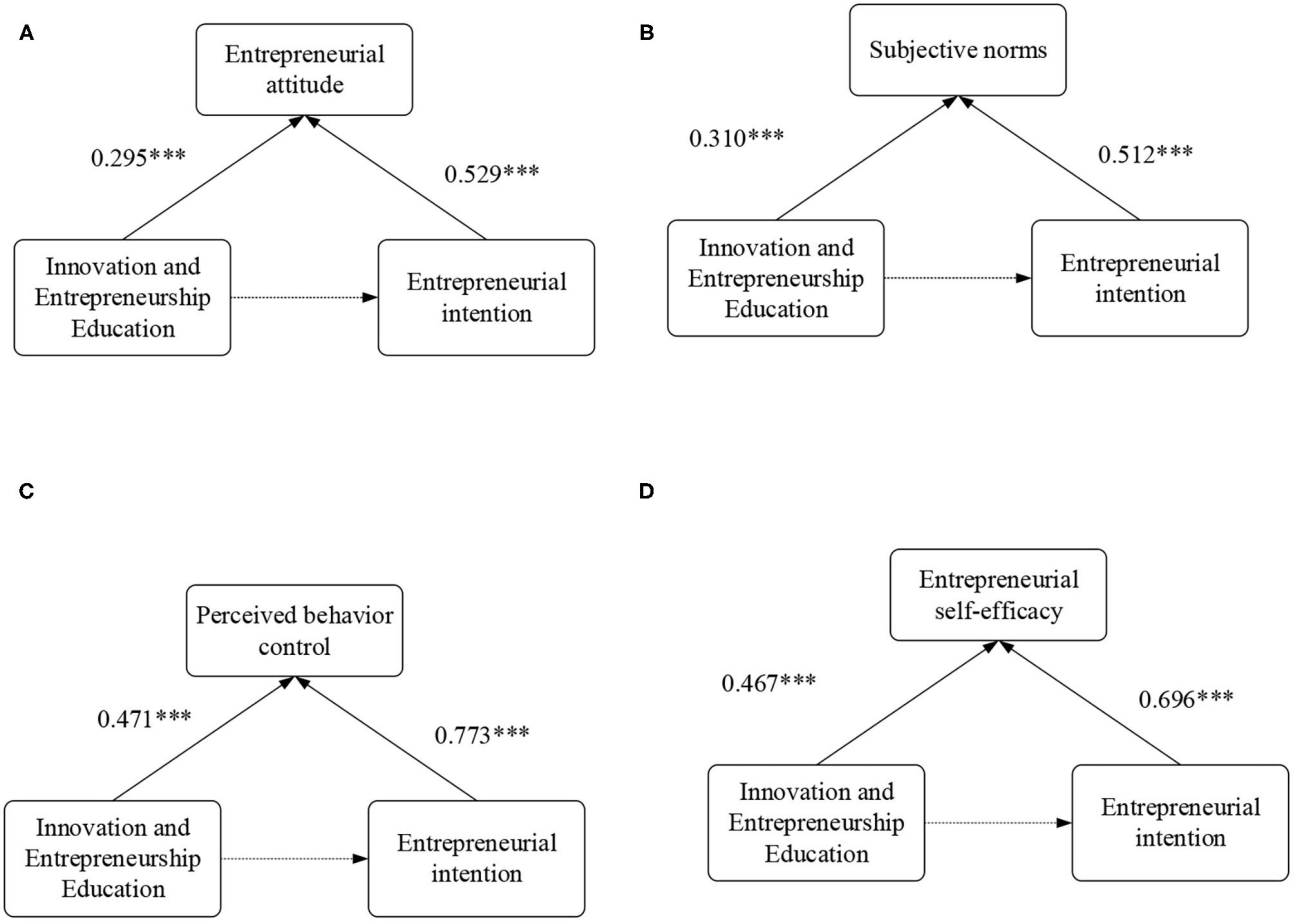
Path analysis of the mediating effect. Figure **(A)** shows the path analysis of entrepreneurial attitude; Figure **(B)** shows the path analysis of main norms; Figure **(C)** shows the path analysis of the perceived behavior control; Figure **(D)** shows the path analysis of entrepreneurial self-efficacy. *** indicates correlation at a significance level of 0.001.

The path analysis of the mediating effect is shown in Figure 7.

Figure 7 shows that entrepreneurial attitude, subjective norms, perceived behavior control, and entrepreneurial self-efficacy play a mediating role in the innovation and entrepreneurship education on college students' entrepreneurial intention. In the processes of “innovation and entrepreneurship education → entrepreneurship attitude” (β = 0.295, *p* < 0.001) and “entrepreneurial attitude → entrepreneurial intention” (β = 0.529, *p* < 0.001), the mediating effect of entrepreneurial attitude is 0.065, and the proportion of the mediating effect of entrepreneurial attitude to the total effect is 0.065/(0.168 + 0.455) = 10.43%, and the direct effect of innovation and entrepreneurship education on college students' entrepreneurial intention is significant (*P* < 0.001). Hypotheses H3 and H4 are verified. In this way, the path analysis and the mediating effect of other variables are analyzed. The proportion of the mediating effect of subjective norms to the total effect is 0.027/(0.168 + 0.455) =4.32%, and the direct effect of innovation and entrepreneurship education on entrepreneurial intention is significant (*P* < 0.001). Therefore, subjective norms play a partial mediating role in the influence of innovation and entrepreneurship education on college students' entrepreneurial intention. The proportion of the mediating effect of perceived behavior control to total effect is 0.302/ (0.168 + 0.455) = 48.51%, and the direct effect of innovation and entrepreneurship education on entrepreneurial intention is significant (*P* < 0.001). Therefore, perceived behavior control plays a partial mediating role in the influence of innovation and entrepreneurship education on entrepreneurial intention. The intermediary effect of entrepreneurial self-efficacy is 0.102, the proportion of the mediating effect of entrepreneurial self-efficacy to the total effect is 0.102/(0.168 + 0.455) = 16.31%, and the direct effect of innovation and entrepreneurship education on entrepreneurial intention is significant (P < 0.001). Therefore, entrepreneurial self-efficacy plays a partial mediating role in the influence of innovation and entrepreneurship education on entrepreneurial intention.

### Model Hypothesis Test Analysis

The AMOS 22.0 software is employed to construct the relationship model of the four variables using the structural equation to further examine the relationships among the variables and test the mediating effect of entrepreneurial self-efficacy and entrepreneurial alertness. After the entrepreneurial experiences of parents and relatives are controlled, a model is established, with college students' entrepreneurial traits as the independent variable, entrepreneurial attitude as the dependent variable, and entrepreneurial self-efficacy and entrepreneurial alertness as the mediating variables. The confirmatory factor analysis shows that the fitting index of the model is excellent (CMIN = 3.253, χ^2^/df = 1.369, GFI = 0.999, CFI = 0.999, TLI = 0.999, NFI = 0.998, RMSEA = 0.062, *P* = 0.254 > 0.05), CMIN represents the chi-square value, χ^2^/df represents the statistics of the similarity between the covariance matrix of the verification sample and the variance matrix, GFI refers to the Goodness-of-fit index, CFI refers to Comparative fit index, TLI refers to Tucker-Lewis index, NFI refers to normal of fit index, RMSEA refers to the Root-mean-square error of approximation, and *P* represents the *P*-value.

The bias-corrected Bootstrap is applied to test the significance of the mediating effect. The original sample is taken as the population of Bootstrap sampling. Through repeated sampling with replacement, 2,000 Bootstrap samples are set in AMOS 22.0, and relevant statistics are obtained. The analysis results are summarized in Table 3.

**Table 3:** Analysis of multiple mediation effects of influencing factors.

**Effects**	**Paths**	**Effect size**	**The proportion in total effect (%)**
Direct effect	Entrepreneurial traits → entrepreneurial attitude	0.07	15.22
Indirect effect 1	Entrepreneurial traits → entrepreneurial self-efficacy → entrepreneurial attitude	0.144	36.91
Indirect effect 2	Entrepreneurial traits → entrepreneurial alertness → entrepreneurial attitude	0.151	38.72
Indirect effect 3	Entrepreneurial traits → entrepreneurial self-efficacy → entrepreneurial alertness → entrepreneurial attitude	0.036	9.15
Total mediating effect		0.39	84.78
Total effect		0.46	100

Data results in Table 3 show the three paths of chained mediating effect: (1) through the mediation path of entrepreneurial self-efficacy, the amount of mediating effect reaches 36.91%. (2) Through the mediation path of entrepreneurial alertness, the amount of mediating effect reaches 38.72%. (3) Through the mediation path of entrepreneurial self-efficacy and entrepreneurial alertness, the amount of mediating effect is 9.15%. Data comparison suggests that the entrepreneurial traits of college students are significantly positively correlated to entrepreneurial attitude; the entrepreneurial self-efficacy of college students is significantly positively correlated to entrepreneurial attitude; the entrepreneurial alertness of college students is significantly positively correlated to entrepreneurial attitude. College students' entrepreneurial self-efficacy plays a mediating role in the relationship between entrepreneurial traits and entrepreneurial attitude, and college students' entrepreneurial alertness plays a mediating role in the relationship between entrepreneurial traits and entrepreneurial attitude. The above results show that the entrepreneurial traits of college students affect their entrepreneurial attitude through the two mediation paths of entrepreneurial self-efficacy and entrepreneurial alertness, revealing a significant correlation between the two (*r* = 0.634, *P* < 0.001). Hence, entrepreneurial alertness and entrepreneurial self-efficacy play a vital role in the relationship between entrepreneurial traits and entrepreneurial attitude. The above results indicate that entrepreneurial self-efficacy and entrepreneurial alertness play multiple chained mediating roles between college students' entrepreneurial traits and entrepreneurial attitude. The above empirical research can verify the research hypotheses proposed.

## Discussion

The research shows that entrepreneurial alertness is positively correlated with entrepreneurial self-efficacy. The higher entrepreneurial alertness is, the higher entrepreneurial self-efficacy is. The significance test shows that the stronger the entrepreneurial attitude is, the higher the entrepreneurial self-efficacy is. That is, the stronger entrepreneurial attitude is, the higher entrepreneurial alertness is. Entrepreneurial traits, entrepreneurial self-efficacy, entrepreneurial alertness, and entrepreneurial attitude are significantly correlated. There is a significant positive correlation between college students' entrepreneurial traits and entrepreneurial attitude; there is a significant positive correlation between college students' entrepreneurial self-efficacy and entrepreneurial attitude; there is a significant positive correlation between college students' entrepreneurial alertness and entrepreneurial attitude. College students' entrepreneurial self-efficacy plays a mediating role in the relationship between entrepreneurial traits and entrepreneurial attitude, and college students' entrepreneurial alertness plays a mediating role in the relationship between entrepreneurial traits and entrepreneurial attitude. The above results show that the entrepreneurial traits of college students affect their entrepreneurial attitude through entrepreneurial self-efficacy and entrepreneurial alertness, and reveal a significant correlation between them (*r* = 0.634, *P* < 0.001). Therefore, entrepreneurial alertness and entrepreneurial self-efficacy play an important role in the relationship between entrepreneurial traits and entrepreneurial attitude. The above results show that college students' entrepreneurial self-efficacy and entrepreneurial alertness play a multiple-chain mediating role between college students' entrepreneurial traits and entrepreneurial attitude. The above empirical research can verify the research hypotheses.

Based on the above results, the following suggestions are proposed: (1) the entrepreneurial intention of college students should be enhanced to promote the improvement of entrepreneurship education in colleges and universities and realize the multiplier effect of entrepreneurship on promoting employment; (2) entrepreneurial practice is strengthened to improve college students' entrepreneurial self-efficacy, promote the entrepreneurial models, interpret college students' entrepreneurial experience and successful experience, provide indirect entrepreneurial success experience for college students, and stimulate college students' entrepreneurial passion and dream; (3) a “heterogeneous” entrepreneurial team is built to improve college students' entrepreneurial awareness. In addition, students should be actively guided to establish a “heterogeneous” entrepreneurial team, and they are encouraged to obtain more information in practice so that their knowledge is enriched, and their entrepreneurial awareness and ability to grasp opportunities should be constantly improved; (4) the classroom teaching methods should be diversified to promote and enhance students' cognitive flexibility, help students establish a variety of entrepreneurial cognitive performance, and improve their ability to use knowledge flexibly; (5) the concept of gender equality in entrepreneurship should be established to improve female college students' entrepreneurial confidence and promote the understanding of female employment and entrepreneurial value.

## Conclusions

Nine hundred and forty-three college students from more than 20 universities are included as research samples to study how entrepreneurial self-efficacy and entrepreneurial alertness affect entrepreneur traits and entrepreneurial attitude. By combining entrepreneurial theories and entrepreneurial spirit, a model of college students' entrepreneurial behavior intentions is established to analyze college students' entrepreneurial intentions and influencing factors of entrepreneurial psychology. Then, hypotheses are verified through questionnaire surveys, and the psychological quality and entrepreneurial attitude of college students are investigated. The empirical analysis results show that entrepreneur self-efficacy and entrepreneur alertness not only play a mediating role but also constitute a chain mediating mechanism between college students' entrepreneurial traits and entrepreneurial attitude. The research results can provide a scientific and theoretical basis for improving college students' entrepreneurial psychological quality and promoting college students' entrepreneurial education. However, in the actual situation, the influencing factors of college students' entrepreneurial intention are complex and changeable, and college students' entrepreneurial psychology is affected by the surrounding complex environment. Therefore, it is difficult to deeply and carefully define the impact of entrepreneurial self-efficacy and entrepreneurial alertness on college students' entrepreneurial intention. Meantime, the questionnaire elements designed are limited, and it is difficult to accurately reveal the interaction between different variables. Therefore, in the future research, more factors will be considered to analyze the impact of entrepreneurial self-efficacy and entrepreneurial alertness on college students' entrepreneurial attitude, thus exploring the role of different variables and influencing factors on entrepreneurial intention.

## Data Availability Statement

The raw data supporting the conclusions of this article will be made available by the authors, without undue reservation.

## Ethics Statement

Ethical review and approval was not required for the study on human participants in accordance with the local legislation and institutional requirements. The patients/participants provided their written informed consent to participate in this study.

## Author Contributions

The author confirms being the sole contributor of this work and has approved it for publication.

## Conflict of Interest

The author declares that the research was conducted in the absence of any commercial or financial relationships that could be construed as a potential conflict of interest.

## Publisher's Note

All claims expressed in this article are solely those of the authors and do not necessarily represent those of their affiliated organizations, or those of the publisher, the editors and the reviewers. Any product that may be evaluated in this article, or claim that may be made by its manufacturer, is not guaranteed or endorsed by the publisher.
